# Neural Substrates of Cognitive Motor Interference During Walking; Peripheral and Central Mechanisms

**DOI:** 10.3389/fnhum.2018.00536

**Published:** 2019-01-09

**Authors:** Emad Al-Yahya, Wala’ Mahmoud, Daan Meester, Patrick Esser, Helen Dawes

**Affiliations:** ^1^School of Rehabilitation Sciences, The University of Jordan, Amman, Jordan; ^2^Movement Science Group, Faculty of Health and Life Sciences, Oxford Brookes University, Oxford, United Kingdom; ^3^Institute for Clinical Psychology and Behavioural Neurobiology, Eberhard Karls Universität Tübingen, Tübingen, Germany; ^4^Faculty of Health and Life Sciences, Centre for Movement, Occupational and Rehabilitation Sciences, OxINMAHR, Oxford Brookes University, Oxford, United Kingdom

**Keywords:** gait control, prefrontal cortex, motor cortex, H-reflex, fNIRS, Parkinson disease, cognitive motor interference

## Abstract

Current gait control models suggest that independent locomotion depends on central and peripheral mechanisms. However, less information is available on the integration of these mechanisms for adaptive walking. In this cross-sectional study, we investigated gait control mechanisms in people with Parkinson’s disease (PD) and healthy older (HO) adults: at self-selected walking speed (SSWS) and at fast walking speed (FWS). We measured effect of additional cognitive task (DT) and increased speed on prefrontal (PFC) and motor cortex (M1) activation, and Soleus H-reflex gain. Under DT-conditions we observed increased activation in PFC and M1. Whilst H-reflex gain decreased with additional cognitive load for both groups and speeds, H-reflex gain was lower in PD compared to HO while walking under ST condition at SSWS. Attentional load in PFC excites M1, which in turn increases inhibition on H-reflex activity during walking and reduces activity and sensitivity of peripheral reflex during the stance phase of gait. Importantly this effect on sensitivity was greater in HO. We have previously observed that the PFC copes with increased attentional load in young adults with no impact on peripheral reflexes and we suggest that gait instability in PD may in part be due to altered sensorimotor functioning reducing the sensitivity of peripheral reflexes.

## Introduction

Walking, although a largely automatic process, is controlled by the cortex, brain stem and the spinal cord; with corrective reflexes modulated through integration of neural signals from central and peripheral inputs throughout the gait cycle (Nielsen, 2003; Yang and Gorassini, 2006; Meester et al., 2014; Minassian et al., 2015). Controlled experiments and studies from pathological populations demonstrate that impairments to any of these systems affect gait control (Axer et al., 2010; Takakusaki, 2017). In particular, gait adaptation to unpredictable environmental constraints is challenged by such impairments, rendering ambulation unsafe and increasing fall risks (Axer et al., 2010; Hamacher et al., 2015). However, whilst much is known about each of the systems independent working, there is less information of the integration of central and peripheral mechanisms during walking. A better understanding of gait control mechanisms in healthy adults and in those with increased risk of falls may inform novel therapeutic approaches.

On the one hand, we know that central motor networks, including the motor, premotor, and prefrontal cortices are active during walking and that additional cognitive tasks can interfere with walking performance particularly in older adults or those with pathology (Suzuki et al., 2004; Al-Yahya et al., 2011; Hamacher et al., 2015). For example, increased prefrontal cortex (PFC) activation under dual task (DT) walking conditions has been repeatedly reported in different groups of participants (Hamacher et al., 2015; Herold et al., 2017). Previously, we have found that DT walking resulted in increased PFC activation in healthy adults and in chronic stroke survivors (Meester et al., 2014; Al-Yahya et al., 2016). In other studies, DT-related increase in PFC activation has been shown in healthy elderly (Holtzer et al., 2015) and in people with Parkinson’s disease (PD) (Maidan et al., 2016; Nieuwhof et al., 2016). One suggested mechanism is through central motor networks influence on spinal reflex circuits via corticospinal inputs to alpha motor-neuron (Knikou, 2008, 2010). In particular, PFC capacity to activate the motor cortex required for task execution (Corp et al., 2013; Fujiyama et al., 2016), as activity in the motor cortex has been reported to be directly involved in leg muscles control during human walking (Petersen et al., 2001).

On the other hand, previous studies show that H-reflex gain can be modulated based on task and environment. For example, it has been reported that H-reflex is attenuated during running and narrow beam walking (Capaday and Stein, 1987; Llewellyn et al., 1990), during DT standing (Weaver et al., 2012), and in the elderly compared to young adults (Chalmers and Knutzen, 2000). Combined, these results suggest that the gain of the H-reflex is reduced, indicating a depressed spinal reflex excitability, in tasks or individuals requiring greater stability (Zehr, 2002). Perhaps, H-reflex is suppressed to prevent excessive reflex activation of motor neurons, and possible instability in the stretch reflex feedback loop, while allowing high levels of afferent signals to continue to supraspinal structures so that these signals can contribute to locomotor activity (Capaday and Stein, 1987; Llewellyn et al., 1990; Hayashi et al., 1992). In support of this hypothesis, it has been suggested that H-reflex suppression in complex postural tasks may reflect more cortical control over the task as well as to prevent unwanted oscillations in postural control (Koceja et al., 1995), and H-reflex is relatively resistance to modulation when conditioning sources are peripheral (Garrett et al., 1999; Ferris et al., 2001), suggesting a central control of reflex modulation while walking (Phadke et al., 2010).

However, brain pathologies, such as stroke or PD, lead to defective utilization of these peripheral inputs by spinal reflex circuits (Dietz, 2002; Hiraoka et al., 2005; Mullie and Duclos, 2014). In addition to the basal ganglion pathology, alterations in a widespread supraspinal locomotor network have been suggested to underlie distinctive gait dysfunctions in people with PD (Amano et al., 2013; Peterson and Horak, 2016). Moreover, impaired modulation of the Soleus H-reflex during standing (Hayashi et al., 1997) and gait initiation (Hiraoka et al., 2005) has been reported in people with PD, which might be attributed to abnormal descending control. Therefore, a better understanding of how peripheral and central mechanism are integrated to modulate spinal circuits may inform effective interventions. To date there have been no studies exploring these mechanisms simultaneously in older healthy adults and those with known gait pathology. In this study in healthy older adults and people with PD we used an additional cognitive task to interfere with the automatic processing occurring during locomotion on a treadmill by introducing a backward counting task (Al-Yahya et al., 2009). We investigated the effect of the additional demands on the PFC and motor cortex activities and on Soleus H-reflex gain alongside gait performance (Suzuki et al., 2004, 2008; McCulloch, 2007). We anticipated that differences in the involvement of peripheral and central mechanisms will emerge between people with PD and healthy older adults and these differences will be relative to the walking task.

## Materials and Methods

This study was approved by Oxford Brookes University Research Ethics Committee (UREC120604). All methods were carried out in accordance with the latest guidelines and regulations of the Declaration of Helsinki. All subjects gave informed, written consent prior to participation.

### Participants

We recruited two groups of participants to take part in this cross-sectional randomized repeated measure study; people diagnosed with Parkinson’s disease (PD) and age similar healthy older adults (HO). General inclusion criteria for all participants were (a) above 50 years old; (b) no named neurological condition other than PD for the experimental group; (c) mentally intact; (d) able to walk safely and continuously on a treadmill for at least 5 min; and (e) able to give written consent. People with PD were included if they were also (a) diagnosed with idiopathic PD as confirmed by a neurologist; (b) taking their prescribed anti-Parkinsonian medication; and (c) fully independent in all activities of daily living as per Hoehn and Yahr scale (Hoehn and Yahr, 1967). Healthy control were basically relatives of PD patients/interested people from local community, or university employee. They were defined healthy as per their own words. Participants were excluded if they had psychiatric comorbidity, clinical diagnosis of dementia or other clinically significant cognitive impairment, and a history of any neurological disorder that could affect their performance (other than PD).

For patients with PD, we conducted all tests within the practical self-reported “ON” medications state (roughly within 1–2 h after medication intake). PD patients were in general moderately physically active per self-report.

### Study Design and Procedure

In order to standardize the assessment, we advised all participants to avoid strenuous exercise 24 h prior to the assessment. Initially, we familiarized participants with the treadmill walking (Woodway ELG 75, Germany) at varying speeds. Thus, for each participant we determined a preferred self-selected treadmill speed, which relatively corresponds to individual’s natural over-ground walking speed (Al-Yahya et al., 2009; Meester et al., 2014), hereinafter referred to as self-selected walking speed (SSWS). We then increased the SSWS by 20% to determine a faster walking (Voloshin, 2000; Meester et al., 2014), hereinafter referred to as fast-walking speed (FWS).

We then introduced participants to the cognitive (i.e., distracting) task. For the cognitive task, we utilized serial subtraction by a given number (i.e., counting backward by 7 s). Serial subtraction is a mental tracking task that loads cognitive resources of the brain that are otherwise engaged in processing attention and working memory (Al-Yahya et al., 2011). This cognitive task is also known to negatively affect walking performance, not only among the elderly and individuals with neurologic disorders but also in healthy young adults (Al-Yahya et al., 2011).

Each participant then completed two walking trials; one at SSWS and the other at FWS in a pseudo random order. Each trial consisted of five blocks of single-task (ST) walking and five blocks of dual-task (DT) walking (i.e., subtracting whilst walking) conditions. For each trial, walking tasks consisted of a 30 s task period repeated 10 times (5 blocks of ST walking and 5 blocks of DT walking) and alternated with rest periods. To avoid anticipation of walking blocks onset, the duration of rest periods ranged from 25 to 45 s in a pseudo-random order. During the DT blocks, participants were asked to count backward in sevens from a random 3-digit number. The instructions before ST and DT blocks were standardized for all participants and they were not given advice as to which task to prioritize during DT-walking. To monitor hemodynamic responses, systematic blood pressure, and heart rate were measured at the beginning and after the end of each trial.

### fNIRS Imaging and Processing

Cortical brain activation was measured with the OxyMon Mk III system (Artinis Medical Systems, Netherlands). The continuous-wave multichannel system uses two wavelengths at 782 and 859 nm to estimate relative changes in the concentration of oxygenated (oxy-Hb) and deoxygenated (deoxy-Hb) hemoglobin, respectively.

In order to estimate PFC task-related activation, two identical plastic holders consisting of two optodes each (one source and one detector) with an inter optode distance of 30 mm were placed on each participant’s forehead using a custom-built spring-loaded holder centered in the area linking Fp1, F3, and F7 and the area linking Fp2, F4, and F8 according to the international 10–20 EEG electrode placement system, which corresponds to the left and right PFC, respectively (Leff et al., 2008; Al-Yahya et al., 2016).

**FIGURE 1:**
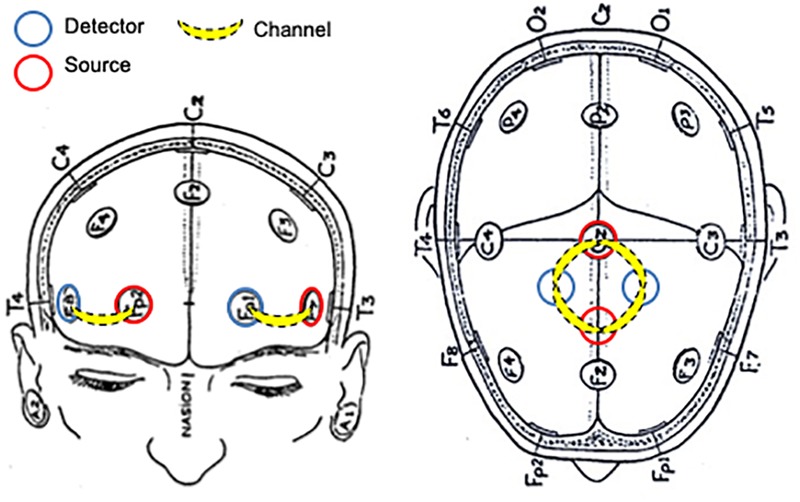
Schematic representation of NIRS probe location.

Additionally, a 4-channel arrangement (two sources and two detectors) was aligned with Cz (Figure 1). The four channels within this arrangement covered the area linking Cz, C3, F3, and Fz, and the area linking Cz, C4, F4, and Fz, which corresponds to left and right sensorimotor cortex (SMC), respectively, including the primary motor cortex (M1) (Okamoto et al., 2004; Koenraadt et al., 2014).

Optical signals were continuously sampled at 10 Hz, and stored according to their wavelength and location, resulting in values for changes in the concentration of oxy-Hb and deoxy-Hb from each channel. Optical data were used to quantify task-related changes of oxy-Hb and deoxy-Hb based on the modified Beer-Lambert law (Delpy et al., 1988).

NIRS data processing was performed using the OxySoft software (version 2.1.6). Standard pre-processing and individual-level statistical analysis was applied (Meester et al., 2014; Al-Yahya et al., 2016). Initially, traces were visually inspected by two investigators independently to identify motion artifacts. Motion artifacts were defined as sudden changes in the amplitude of the NIRS signals (oxy-Hb and deoxy-Hb) that were much larger than the expected changes and that appeared in several channels, while missing signals were characterized by flat-line appearing traces of oxy-Hb and/or deoxy-Hb changes. Blocks with motion artifacts were excluded from further analysis. To remove high frequency noise such as cardiac pulsation, NIRS signals were then low-pass filtered at a 0.67 Hz cut off frequency using a customized LabVIEW program (National Instruments, Austin, TX, United States). A moving average filter with a width of 4 s was then used to smooth the signal. Block averages of the task plus rest repetitions were calculated and the middle 10 s of each task and rest periods used for statistical analyses. To identify channels exhibiting task-related activation, average concentration changes were compared, by means of *t*-tests for all tasks to the average baseline-corrected on a block-by-block basis by subtracting the mean intensity of the 5 s preceding trial onset (i.e., last 5 s of rest block) from the overall task block. Active channels were defined as statistically different relative to baseline. For each task, signals from all repeats were averaged, and data from active channels were used for subsequent statistical analyses.

### H-Reflex Measurement and Gain Calculation

**FIGURE 2:**
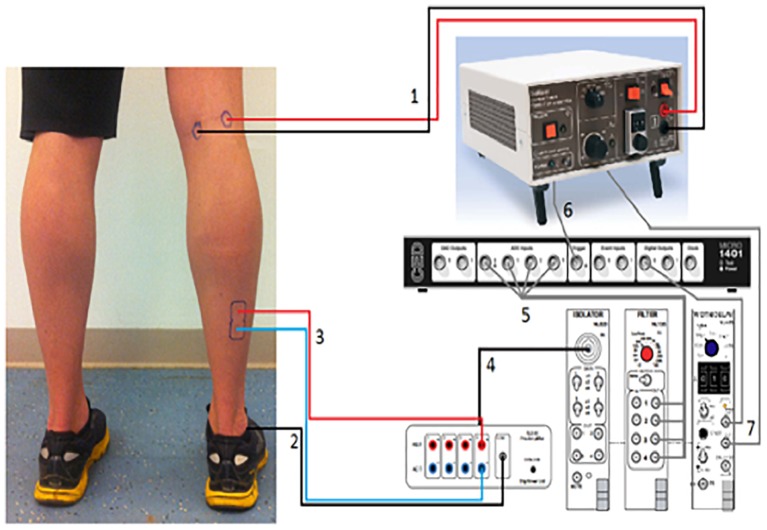
H-reflex measurement setup. (1) The cables from the stimulator to the Tibial nerve in the popliteal fossa. (2) Reference electrode going to the lateral Malleolus. (3) The cables from the EMG-amplifier going to the Soleus. (4) The EMG-signal going to isolator which is connected with the filter. (5) The filtered EMG-signal is going to the CED-box. This box is connected with the pc. (6) The signal from the stimulator is also sent to the CED-box. (7) “Width-tool,” which sends the signal to the CED-box. With a pc the signals from the CED-box can be viewed in the “Signal Software.”

H-reflexes were elicited in the right Soleus muscle (SOL) as described before (Meester et al., 2014). We utilized a constant current high voltage stimulator (Digitimer Ltd., DS7A, United Kingdom) to elicit H-reflexes and M-waves (Figure 2). We recorded H-reflex recruitment curves while participants were standing. In order to determine the intensity which would elicit 20–25% of Mmax (Simonsen and Dyhre-Poulsen, 1999; Phadke et al., 2010) we measured Hmax and Mmax. A footswitch (Odstock Medical Ltd., United Kingdom) under the participant’s right heel was used to synchronize H-reflex with the mid stance [30% (Hughes and Jacobs, 1979)] phase of the gait cycle. To prevent depression of the H-reflex and subject anticipation of the reflex, stimulations were given every 4–6 heel strikes; corresponding to an inter-stimulus-time (ISI) of 4–5 s, which is known to be long enough to measure consecutive H-reflexes (Knikou and Taglianetti, 2006; Jeon et al., 2007).

Ag–AgCl electrodes were placed on the muscle belly and as a stimulating electrode on the Tibial nerve (Konrad, 2005). The cathode was placed in the popliteal fossa with the anode at a distance of 2 cm medial to the cathode (Figure 2). We located the nerve using small moveable electrodes, before positioning the actual stimulation electrodes, which were secured with Velcro tape to prevent slippage during locomotion. EMG leads were attached to the leg and upper body to reduce movement artifacts and prevent subjects from tripping.

Data acquisition and initial analysis were performed using Signal software (CED Signal 3.09, United Kingdom). EMG signals were pre-amplified and high passed filtered at 42.5 Hz (NL844; Digitimer). Signals were then low pass filtered at 200 Hz (NL135; Digitimer) before H-reflexes were sampled at 1000 Hz (Tokuno et al., 2007). A customized LabVIEW program (National Instruments, Austin, TX, United States) was used to automatically detect the H-reflex and calculate its peak-to-peak amplitude.

We calculated H-reflex gain as described previously (Capaday and Stein, 1987; Edamura et al., 1991). For each measurement at 30% of the gait cycle, we calculated the average of the rectified EMG during the time frame. Then, we plotted a data point for each time frame with the corresponding H-reflex amplitude on the *y*-axis and the averaged rectified EMG on the *x*-axis. We fitted a linear least-square regression model to the data points at each walking task for each subject. Then, we calculated the slopes of the H-reflex amplitudes versus averaged background EMG regression lines.

### Gait Measurement

Step time was measured using an inertial measuring unit (Philips, Eindhoven, Netherlands) comprising a tri-axial accelerometer, gyroscope and magnetometer placed on the projected center of mass located over the fourth lumbar vertebrae (Esser et al., 2012). Post-processing and analysis was performed using a customized prewritten program in LabVIEW (National Instruments, Austin, TX, United States). Step time was then estimated according to the inverted pendulum gait model (Esser et al., 2009), and variability of step time was estimated using the standard deviation.

### Statistical Analysis

Statistical analysis was performed using IBM-SPSS 25.0 (IBM SPSS, Inc., Armonk, NY, United States). At first, we performed descriptive. statistical analysis on demographics (Tables 1, 2). Then, we analyzed behavioral (i.e., step time and step time variability) and physiological (i.e., H-reflex gain) gait measures by conducting a mixed-design analysis of variance (ANOVA) with task (two levels; ST-walking and DT-walking) and speed (two levels; SSWS and FWS) as the independent within-subjects variables for each measure separately, and participants’ group (two levels; PD and HO) as the between-subject factor.

**Table 1:** Participants characteristics.

	*N*	Females	Age^∗^ (mean ± SD)	SSWS^∗^ (mean ± SD) m/s	FWS^∗^ (mean ± SD) m/s
Old	22	16	59.5 ± 6.8	1.04 ± 0.19	1.24 ± 0.23
PD	29	13	66.3 ± 5.9	0.89 ± 0.21	1.06 ± 0.26

*^∗^Independent samples *t*-test (two-tailed) revealed significant differences between groups (*p* < 0.05).*

**Table 2:** Parkinson’s disease group detailed characteristics.

Barthel index score^1^: median (range)	20 (19–20)
Tripping without falling: number (%)	10 (34.5%)
Falling: number (%)	7 (24%)
FOG^2^: number (%)	7 (24%)
HY^3^ stage (“ON meds”): number (%)	
HY = 1	20 (70%)
HY = 2	9 (30%)
HY = 3	0
HY = 4	0
HY = 5	0
MDS-UPDRS^4^ (“ON meds”) mean (SD)	
Part I (0–52)	8.6 (4.5)
Part II (0–52)	8.25 (5.1)
Part III (0–132)	16.7 (10)
Part IV (0–24)	1.65 (2.6)

*^*1*^A scale for activity of daily living (Mahoney and Barthel, 1965). ^*2*^Freezing of gait. ^*3*^Hoehn-Yahr Classification of Disability Scale (Hoehn and Yahr, 1967): (1) Absent or minimal unilateral disability; (2) Minimal bilateral or midline involvement with intact balance; (3) Unsteadiness when turning or rising with some activities restrictions, patients are independent; (4) Severe symptoms and possible walking with assistance; (5) Confined to bed or wheelchair. ^*4*^Unified Parkinson’s Disease Rating Scale, a measure of disease progression (Goetz et al., 2008).*

For the NIRS data statistical analysis, the average of relative changes in oxy-Hb and deoxy-Hb concentrations of combined activated blocks was calculated for each task and region (left PFC, left M1, and right M1). Initially, we intended to include the site (left vs. right PFC) as another factor in our NIRS analysis. Due to unforeseen technical reasons, the NIRS signals from the right PFC showed very low gain for almost all participants and, therefore, it was not included in the final analysis. For each region (i.e., left PFC, right M1, and Left M1) data from all participants were analyzed by conducting a mixed-design ANOVA with task (two levels; ST-walking and DT-walking) and speed (two levels; SSWS and FWS) as the independent within-subjects variables while participant group (two levels) was the between-subjects factor. For all statistical tests, alpha level was set at 0.05 *a priori* and SPSS-generated Bonferroni adjusted *P*-values are quoted.

## Results

A total number of 51 participants were recruited and evaluated according to the protocol; 29 individuals with PD and 22 HO adults. PD patients were significantly older and walk slower than HO at both speeds (Table 1). Due to technical difficulties, however, we could not obtain the full data set (gait data, H-reflex, and NIRS data) from all of the participants. In particular, NIRS data from the right PFC were missing for all participants as the signal to noise ratio was very low.

All PD participants were independent in their activities of daily living (Table 2), and the majority of them (70%) had a unilateral involvement according to the Hoehn and Yahr scale. The detailed characteristics of PD participants are summarized in Table 2.

### Behavioral Gait Measures

We first examined whether counting while walking on the treadmill would interfere with gait pattern at either of the walking speed. Average and variability of step time are summarized in Table 3. For step time, mixed-design ANOVA revealed no significant main effect of task [*F*(1,43) = 0.79, *p* = 0.38] or group [*F*(1,43) = 0.11, *p* = 0.74]. However, mixed-design ANOVA revealed a significant main effect of speed [*F*(1,43) = 4.22, *p* = 0.04, η^2^ = 0.089]. Pairwise comparisons revealed that speed effect was only significant for HO group under ST (*p* < 0.001) and DT (*p* = 0.033) conditions (Table 3).

**Table 3:** Gait measurement results (Mean ± SD).

	Old		PD	
	ST	DT	ST	DT
**SSWS**				
Step time (ms)^∗^	**547 ± 69**	**553 ± 76**	530 ± 50	529 ± 82
Step time variability	81.4 ± 80	68.5 ± 72	98 ± 85	94.7 ± 99
**FWS**				
Step time (ms)^∗^	**508 ± 39**	**527 ± 43**	529 ± 70	523 ± 94
Step time variability	44.7 ± 31	50.2 ± 50	103 ± 134	93.9 ± 100

*^∗^Repeated measure ANOVA revealed a significant (*p* < 0.05) main effect of speed on step time. Significant pairwise comparisons (*p* < 0.05) between SSWS and FWS for both tasks are highlighted in bold and appeared only in the old group.*

For step time variability, there were no significant main effects of task [*F*(1,43) = 0.68, *p* = 0.41], speed [*F*(1,43) = 0.97, *p* = 0.33], or group [*F*(1,43) = 2.59, *p* = 0.12]. For both step variables we measured, mixed-design ANOVA revealed no significant interactions between task, speed, and group.

### Physiological Gait Measures

**FIGURE 3:**
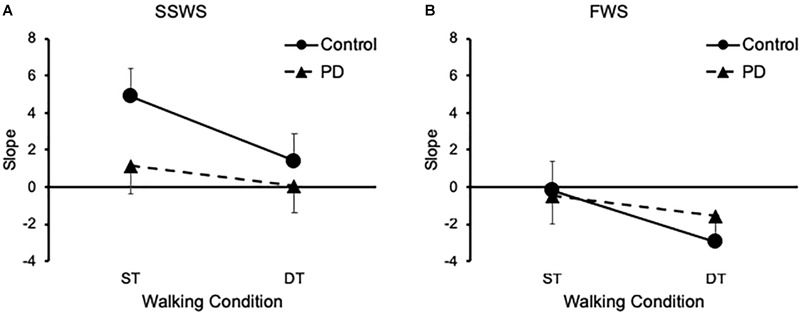
Groups results of the Soleus H-reflex gain. **(A)** Means and standard errors of linear regressions slopes at SSWS. **(B)** Means and standard errors of linear regressions slopes at FWS. PD, Parkinson disease; SSWS, self-selected walking speed; FWS, fast walking speed; ST, single task; DT, dual task.

Next we examined the effects of task, speed, and group on H-reflex gain. Our findings are illustrated on Figure 3. Mixed-design ANOVA revealed a significant main effect of task [*F*(1,26) = 30.489, *p* < 0.001, η^2^ = 0.540] on H-reflex gain, which decreased during DT-walking compared with ST-walking for both groups and at the two walking speeds (Figure 3). There was no significant main effect of either speed [*F*(1,26) = 2.978, *p* = 0.057] or group [*F*(1,26) = 1.106, *p* = 0.303] on H-reflex gain. However, there was a significant interaction between task and group [*F*(1,26) = 7.324, *p* = 0.012, η^2^ = 0.220]. Independent samples *t*-test (two-tailed) revealed that OH participants exhibited larger H-reflex gain than PD participants (*p* = 0.045) only while walking under ST condition and at SSWS (Figure 3).

### Cortical Activations

**FIGURE 4:**
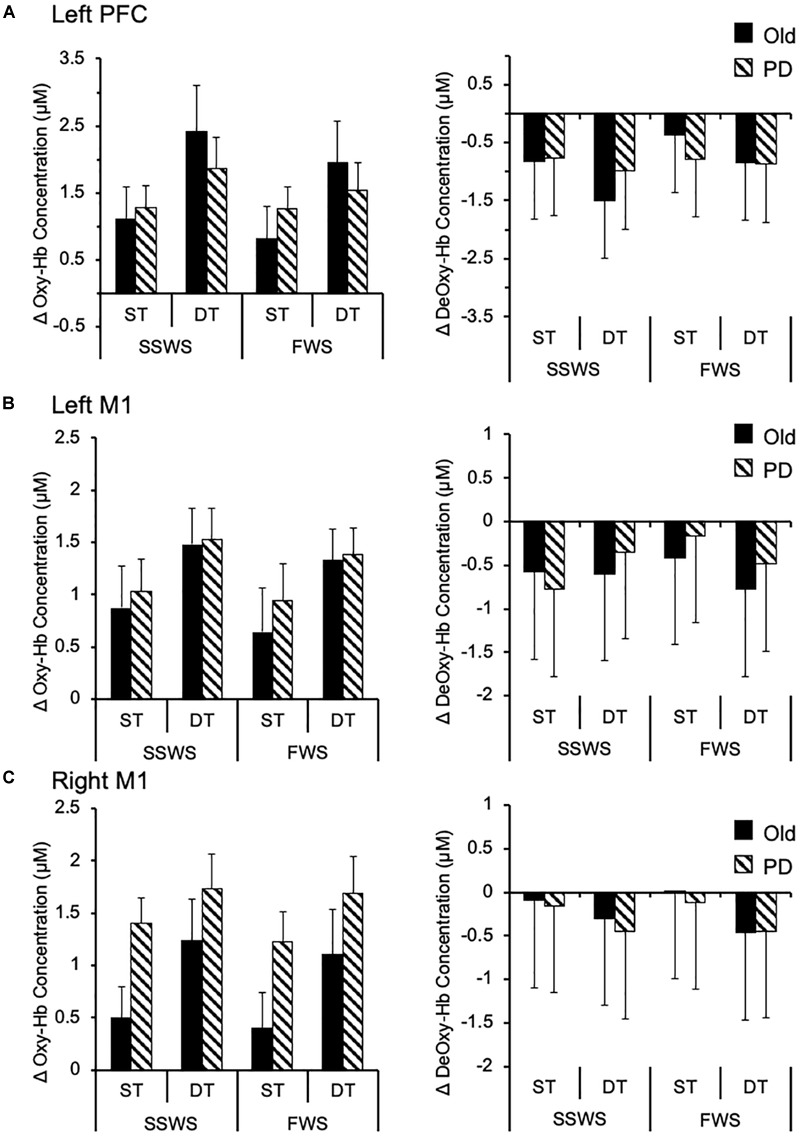
Cortical activation while walking. Group data (means and standard errors) of task-related changes in oxy-Hb (left side) and deoxy-Hb (right side) concentrations while walking under different conditions, in **(A)** left prefrontal cortex, **(B)** left motor cortex, and **(C)** right motor cortex. oxy-Hb, oxygenated hemoglobin; deoxy-Hb, deoxygenated hemoglobin; M1, motor cortex; PFC, prefrontal cortex; PD, Parkinson disease; SSWS, self-selected walking speed; FWS, fast walking speed; ST, single task; DT, dual task.

Finally, we examined the effects of task, speed, and group on cortical brain activation. In particular, we analyzed relative concentration changes of oxy-Hb and deoxy-Hb in left PFC, left M1, and right M1. Results are illustrated on Figure 4 and summarized in Table 4. For the left PFC, mixed-design ANOVA revealed a significant main effect of task on oxy-Hb relative concentration changes [*F*(1,17) = 12.945, *p* = 0.003, η^2^ = 0.648], but not on deoxy-Hb [*F*(1,17) = 4.137, *P* = 0.061, η^2^ = 0.304]. Also, there were no significant main effects of speed or group on either oxy-Hb or deoxy-Hb (Figure 4A). There was a significant main effect of task on oxy-Hb [*F*(1,17) = 31.307, *p* > 0.001, η^2^ = 0.423] and deoxy-Hb [*F*(1,17) = 7.428, *p* = 0.014, η^2^ = 0.305] in left M1, with no significant main effect of either group or speed (Figure 4B). Similarly, there was a significant main effect of task on oxy-Hb [*F*(1,17) = 12.45, *p* = 0.003, η^2^ = 0.480], and deoxy-Hb [*F*(1,17) = 7.45, *p* = 0.014, η^2^ = 0.228] in right M1, with no significant main effect of either speed or group (Figure 4C). For all measured cortical areas, there were no significant interactions between task, speed, and group.

**Table 4:** Brain activation as measured by relative concentration changes (in μM) in Oxy-Hb and DeOxy-Hb (Mean ± SE).

Area	SSWS		FWS	
	ST	DT	ST	DT
**Left M1**				
*Oxy-Hb^∗^*				
**Old**	**0.876 ± 0.394**	**1.484 ± 0.345**	**0.641 ± 0.419**	**1.330 ± 0.301**
**PD**	**1.037 ± 0.336**	**1.529 ± 0.294**	**0.941 ± 0.358**	**1.381 ± 0.257**
*DeOxy-Hb^∗^*				
**Old**	-0.575 ± 0.258	-0.599 ± 0.28	-**0.412 ± 0.251**	-**0.775 ± 0.26**
**PD**	-**0.018 ± 0.22**	-**0.346 ± 0.239**	-0.161 ± 0.214	-0.484 ± 0.222
**Right M1**				
*Oxy-Hb^∗^*				
**Old**	0.501 ± 0.291	1.242 ± 0.39	**0.399 ± 0.338**	**1.107 ± 0.422**
**PD**	**1.400 ± 0.248**	**1.731 ± 0.333**	**1.223 ± 0.288**	**1.684 ± 0.36**
*DeOxy-Hb^∗^*				
**Old**	-0.095 ± 0.104	-0.302 ± 0.199	0.004 ± 0.192	-0.467 ± 0.249
**PD**	-**0.156 ± 0.089**	-**0.450 ± 0.17**	-0.117 ± 0.163	-0.447 ± 0.212
**Left PFC**				
*Oxy-Hb^∗^*				
**Old**	1.104 ± 0.49	2.422 ± 0.688	**0.814 ± 0.478**	**1.952 ± 0.62**
**PD**	**1.277 ± 0.33**	**1.872 ± 0.464**	1.266 ± 0.322	1.533 ± 0.418
*DeOxy-Hb*				
**Old**	-0.824 ± 0.364	-1.501 ± 0.501	-0.363 ± 0.333	-0.838 ± 0.499
**PD**	-0.759 ± 0.245	-0.987 ± 0.338	-0.783 ± 0.225	-0.872 ± 0.338

*Repeated measure ANOVA revealed a significant (^∗^*p* < 0.05) main effect of task on cortical activation. Significant pairwise comparisons (*p* < 0.05) between ST and DT for both groups and speeds are highlighted in bold.*

## Discussion

In the present study we investigated neuronal control mechanisms affecting gait performance in people with PD and a group of healthy older adults (HO). We measured the effect of an additional cognitive task and increased walking speed on the pre-frontal and motor cortex activation, and the Soleus H-reflex gain alongside behavioral gait performance. Under DT conditions, as we expected, we observed increased cortical activation in PFC and M1 for both groups and speeds. Whilst, H-reflex gain decreased with the additional cognitive load and somehow increased speed for both groups, H-reflex gain was lower in PD compared to HO only while walking under ST condition at SSWS. Concerning behavioral gait measures, DT interference during walking at both speeds was comparable for PD and HO controls. All in all, these observations show hitherto unreported integration of central and peripheral mechanisms of gait control in PD and HO. We propose that attentional load in the PFC excites the M1, which in turn increases inhibition on H-reflex activity during walking and reduces the activity and sensitivity of peripheral reflex during the stance phase of gait. Importantly this effect on sensitivity was greater in HO. We have previously observed that the PFC appears to be able to cope with increased attentional load in young adults with no impact on peripheral reflexes (Meester et al., 2014), and we suggest that gait instability in PD may in part be due to altered sensorimotor functioning reducing the sensitivity of peripheral reflexes.

Safe, independent, and goal-directed gait is crucial for daily living and relies on complex neuronal networks encompassing supraspinal and spinal structures of the CNS. On the one hand, supraspinal structures usually participate in appropriate planning and precise control of movement (Drew and Marigold, 2015). Thus, they are indispensable for adapting gait to the behavioral goals. On the other hand, spinal circuits modulate corrective reflexes throughout the gait cycle by integrating neural signals from peripheral as well as central inputs (Nielsen, 2003; Yang and Gorassini, 2006; Meester et al., 2014; Minassian et al., 2015).

Mounting evidence from neuroimaging studies suggests that stable and goal-directed gait requires the activation of both cortical and subcortical structures of the brain (Hamacher et al., 2015; Herold et al., 2017). In particular, two cortical networks have been proposed; a direct and an indirect pathway (la Fougère et al., 2010; Zwergal et al., 2012, 2013). The direct pathway is potentially involved in the automatic control of gait via M1, cerebellum, and spinal cord. The indirect pathway emerges in challenging situations (i.e., those that require cognitive as opposed to automatic gait control) and additionally involves prefrontal and premotor areas (Hamacher et al., 2015; Herold et al., 2017). Therefore, gait control entails a subtle balance between automatic and executive control processes, which is dependent upon task demands and individual’s ability (Clark, 2015). Findings from our study support this notion; we observed increased PFC and M1 activation under DT walking compared to ST in both groups.

Increased PFC activation under DT walking conditions has been repeatedly reported in different groups of participants (Hamacher et al., 2015; Herold et al., 2017). Previously, we have found that DT walking resulted in increased PFC activation in healthy adults and in chronic stroke survivors (Meester et al., 2014; Al-Yahya et al., 2016). In other studies, DT-related increase in PFC activation has been shown in healthy elderly (Holtzer et al., 2015) and in people with PD (Maidan et al., 2016; Nieuwhof et al., 2016). Although healthy young adults responded to additional cognitive load with increased PFC activation (Meester et al., 2014), this was not associated with altered gait parameters. Whereas, a greater increase in PFC activation observed from ST to DT walking was related to a greater change in motor performance in stroke survivors (Al-Yahya et al., 2016). Similarly, a negative association between PFC activation and gait performance was observed in healthy elderly (Holtzer et al., 2016) and in people with PD (Maidan et al., 2016). In contrast, walking at different steady state speeds did not considerably affect PFC activation (Suzuki et al., 2004; Meester et al., 2014), which is similar to what we have found in this study. This is presumably due to the ability of brain stem and spinal circuits within the direct pathway to automatically adjust gait pattern without a substantial need for executive control (Clark, 2015). Taken together, these findings alongside ours are in line with a recently suggested model of gait control (Clark, 2015; Gramigna et al., 2017). This model proposes that when the automatic execution of gait is compromised, such that by normal aging, pathology of the CNS, or episodic cognitive load, individuals may compensate by switching to cognitive control, in particular executive control in the PFC.

Performing complex motor tasks, as opposed to simple ones, require additional activations in subcortical and cortical regions other than the PFC (Godde and Voelcker-Rehage, 2010). For example, consistent findings from NIRS imaging studies show gait related changes in motor and somatosensory regions (Suzuki et al., 2004; Kurz et al., 2012; Koenraadt et al., 2014). In the present study, we observed DT related increase in M1 activation in both groups during walking at different speeds.

Successful and adaptive control of motor performance requires not only excitatory, but also inhibitory functioning of neural circuits within motor area (Beck et al., 2008; Beck and Hallett, 2011). Recent TMS studies show that older adults exhibited decreased ability to inhibitory function within motor areas as compared to younger adults (Fujiyama et al., 2009), and suggest a significant relationship between motor performance under DT conditions and reduction in inhibitory control within motor areas (Fujiyama et al., 2012a,b). It is likely that the additional task under DT conditions results in under activation of inhibitory circuits within motor areas (Corp et al., 2013), rather than increased activation of the excitatory circuits. Therefore, the observed DT related increase in M1 activation could be attributed to disinhibition, rather than excitation, of motor area.

**FIGURE 5:**
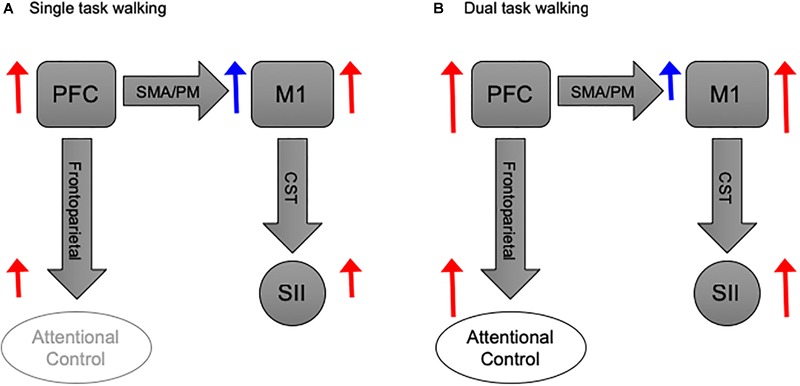
Basic schematic illustration of the functional coupling between PFC and M1 during walking. **(A)** PFC activation during ST walking is devoted to inhibitory modulation of M1 activity via SMA/PM pathways. While PFC involvement in attentional control is tentative, M1 exerts excitatory modulation over SII via CST. **(B)** PFC activation during DT walking, albeit increased, is primarily devoted to attentional control. Thus, its inhibitory effect on M1 becomes less, and the later exerts a more substantial excitatory modulation over SII. Block arrows represent anatomical pathways. Red and blue arrows donate excitatory and inhibitory modulations, respectively. Sizes of blue and red arrows are relative to the proposed activation level. PFC, prefrontal cortex; M1, motor cortex; SMA, sensorimotor area; PM, premotor area; SII, spinal inhibitory interneurons; CST, corticospinal tract.

Furthermore, we observed a considerable level of PFC activation during ST walking relative to baseline (i.e., standing). This may reflect a successful mechanism to compensate for loss of automaticity in PD and HO adults (Seidler et al., 2010), by exerting efficient executive control of gait (Clark, 2015). However, under challenging conditions this compensatory mechanism becomes less efficient, and overloading emerges leading to failure in gait adaptation (Maidan et al., 2017). In fact, the PFC modulates neuronal activity in cortical and subcortical motor structures (Fuster, 2015). Therefore, we propose that during DT walking the PFC becomes otherwise occupied with the executive control of concurrent tasks; thus, rendered less able to modulate its inhibitory control over motor area (Figure 5). However, this last notion requires further investigation.

Previous studies show that the Soleus H-reflex decreases with increased postural task difficulty (Stein and Capaday, 1988; Koceja et al., 1993; Tokuno et al., 2009), such as walking under DT conditions (Weaver et al., 2012). This reduction might reflect a strategy to prevent redundant stretch reflex from causing further postural instability yet permitting proprioceptive signals to reach supraspinal structures (Capaday and Stein, 1987; Llewellyn et al., 1990). Therefore, task-dependent modulation of H-reflex reflects adaptive control of stretch reflex according to task demands (Capaday, 2002). In agreement with this notion, we observed that the Soleus H-reflex gain decreased with the additional cognitive load relative to ST walking.

However, other studies report no modulation of the Soleus H-reflex under DT conditions (Baudry and Gaillard, 2014; Meester et al., 2014). It is worth noting that while participants in Baudry and Gaillard study (Baudry and Gaillard, 2014) were young and elderly adults, and participants in Meester et al’s (2014) study were healthy young adults, neither of the studies measured the Soleus H-reflex gain.

Whilst ascending and descending neural signals can potentially modulate H-reflex (Misiaszek, 2003), available evidence suggests that task-dependent modulation of H-reflex is mediated by descending signals from supraspinal structures (Pijnappels et al., 1998; Haridas et al., 2005), in particular sensory-motor cortical activity (Wolpaw, 2007; Thompson and Wolpaw, 2014, 2015). The observed reduction in H-reflex gain, together with the increased activation of M1 under DT conditions, suggests a cortical origin of H-reflex modulation.

Finally, our observation that H-reflex gain at SSWS was more prominent in HO adults suggests that at steady state walking speed HO adults retain the ability to utilize these adaptive features of spinal circuits, while people with PD do not. However, the additional cognitive load at FWS not only changed the task-demands but also challenged recurrent sensory feedback, thereby rendering such input unreliable affecting stability during gait and potentially stopping normal propulsory peripheral gait inputs (Zehr and Duysens, 2004).

### Methodological Considerations

The interpretation of our results should be considered in light of the following methodological concerns, however. Firstly, due to unforeseen technical reasons, the NIRS signals from the right PFC showed very low gain for almost all participants, which limited our ability to assess brain activation in the right side of PFC. Considering that PFC hemispheric asymmetry has recently been advocated (Eggenberger et al., 2016), we could not rule out whether task-related increased activation was exclusive to the left PFC, or whether PFC asymmetry while walking would be affected by task complexity or individual’s capability. In addition, while NIRS imaging is increasingly advocated to study cortical activations in gait control (Perrey, 2014; Hamacher et al., 2015; Gramigna et al., 2017; Herold et al., 2017), it has modest spatial resolution relative to fMRI. In the present study, our aim was to measure M1 relative activation, yet the recorded signals might reflect activities in M1 alongside adjacent sensorimotor areas. We did not use short separation channels for NIRS so as to control for the effect of superficial hemodynamic from skin (Gagnon et al., 2012; Kirilina et al., 2012). Therefore, we cannot utterly rule out the potential effect of this superficial contamination.

Another limitation concerns treadmill usage. Whilst gait kinematics have been shown to be similar between treadmill and overground walking in healthy adults (Lee and Hidler, 2008; Parvataneni et al., 2009), treadmill use might have posed a constraint, as it did not allow for proper adaptive mechanisms of gait adaptation, by forcing individuals not to adjust walking velocity between ST and DT conditions. However, treadmill use may explain why, counter intuitively, in previous reports PD showed increased PFC during usual over ground walking compared to healthy older adults (Maidan et al., 2016). Treadmill might have been utilized by PD patients as a cue that provided basic rhythm of walking, bypassing the defective basal ganglia in PD. This is supported by the increasing utilization of cues in rehabilitations of Parkinson’s gait (Lohnes and Earhart, 2011; Luessi et al., 2012). An external regulator of gait might have decreased the load on attentional resources (Peterson and Smulders, 2015) that are otherwise used in over-ground walking and rather freed cortical resources to deal with the DT leaving treadmill to guide motion.

Other potential explanations for the lack of between group differences in brain activation might be that HO walked on the treadmill faster than PD at both speeds. Second, PD patients were mostly at initial stages of PD with no obvious or minimal unilateral physical impairments and intact balance. Thirdly, patients were tested “on medication”, that is when the symptoms associated with PD are at the minimal.

Finally, we have not measured the counting task performance under control conditions, which might have changed relative to DT ones. Therefore, this study cannot infer any absolute amount of attention or a mechanism (Fraizer and Mitra, 2008). We rather utilized the serial subtraction task as an attentional distraction to load the system as it has been suggested to do so more effectively than other secondary tasks (Al-Yahya et al., 2011). Presumably by taxing higher-level executive functioning more than low-level divided attention processes (Walshe et al., 2015).

## Conclusion

All in all, our findings provide direct evidence, for the first time, for the integration of central and peripheral control mechanisms during treadmill walking in HO adults and in people with PD. They suggest that the sensorimotor system utilizes different strategies to retain dynamic stability while walking, which could be achieved through adaptive regulation of spinal reflex circuits. This essential adaptive regulation entails a delicate balance between supraspinal, spinal, and peripheral input to spinal circuits, and it is dependent on task demands and individual’s capabilities.

## Data Availability Statement

Data supporting authors’ conclusions will be available by the authors upon request without undue reservation to interested researchers.

## Author Contributions

EA-Y conceived the project, designed the protocol, analyzed the data, performed the statistical analysis, prepared the Figures 3–5, and wrote the manuscript. WM collected that data, analyzed the data, performed the statistical analysis, and reviewed the manuscript. DM designed the protocol, prepared the Figure 1, and reviewed the manuscript. PE supervised the data collection, prepared the Figure 2, and reviewed the manuscript. HD conceived, supervised, and designed the protocol, acted as sponsor of the study, and reviewed the manuscript. EA-Y and HD had full access to all the data in the study and take responsibility for the integrity of the data and the accuracy of the data analysis. All authors gave final approval for submission.

## Conflict of Interest Statement

The authors declare that the research was conducted in the absence of any commercial or financial relationships that could be construed as a potential conflict of interest.
